# Construct validity, responsiveness and minimal important difference of the cumulated ambulation score in older adults with hip fracture in sub-acute rehabilitation facility

**DOI:** 10.1177/02692155241249351

**Published:** 2024-04-24

**Authors:** Ilaria Arcolin, Marica Giardini, Stefano Corna, Francesco Sartorio, Marco Caligari, Marco Godi

**Affiliations:** 1 Department of Physical Medicine and Rehabilitation, Istituti Clinici Scientifici Maugeri IRCCS, Institute of Veruno, Gattico-Veruno, Italy; 2 Department of Scientific Research Campus LUdeS Lugano (CH), Off-Campus Semmelweis University of Budapest, Budapest, Hungary; 3 Integrated Laboratory of Assistive Solutions and Translational Research (LISART), Istituti Clinici Scientifici Maugeri IRCCS, Institute of Pavia, Pavia, Italy

**Keywords:** Aged, hip fractures, patient-reported outcome measures, psychometrics, rehabilitation

## Abstract

**Objective:**

To assess the construct validity, responsiveness and minimal important difference of the cumulated ambulation score in patients with hip fracture in sub-acute rehabilitation facility.

**Design:**

Observational, prospective, monocenter, cohort study.

**Setting:**

Rehabilitation Institute.

**Participants:**

456 older adults with hip fracture (≥65 years) admitted for inpatient rehabilitation.

**Main outcome measures:**

Cumulated ambulation score, functional independence measure and functional ambulation category were collected at admission and discharge. Construct validity and responsiveness were assessed through hypothesis testing and minimal important difference was determined using the anchor-based method; floor and ceiling effects were also assessed.

**Results:**

The cumulated ambulation score showed strong correlations with the functional independence measure and functional ambulation category scores at both admission and discharge, satisfying all the hypotheses for construct validity. The effect size of cumulated ambulation score was 1.63. Changes in cumulated ambulation score had a moderate-to-strong correlation with changes of other instruments and were able to discriminate patients improved from those not-improved, and patients classified as independent ambulators from those dependent. A ceiling effect was found only at discharge. The estimated minimal important difference was 2 points.

**Conclusions:**

The cumulated ambulation score showed high levels of construct validity and responsiveness according to the hypothesis testing. A two points improvement at the end of rehabilitation was found to be clinically important in people with hip fracture in the sub-acute phase. The ceiling effect found at discharge suggested the limitation of the scale in assessing people with a partially recovered autonomy in performing postural changes and gait.

## Introduction

Hip fractures have become a worldwide health problem, resulting in a reduction of mobility and in an increase of mortality rate.^1^ Identifying a reliable and valid measures of mobility status is necessary to monitor and organise the patient's care pathway. The Clinical Practice Guidelines for the physical therapy management of older adults with hip fracture^2^ and a recent systematic review^1^ recommend the use of the cumulated ambulation score for assessing the basic mobility status of patients in both acute and post-acute clinical settings. The cumulated ambulation score is a measure that evaluates patient's mobility independence in three basic activities: getting in and out of bed, sit-to-stand from a chair and walking.^3,4^ It has demonstrated a high level of test–retest and inter-rater reliability, content and construct validity and the capability to predict factors such as length of hospital stay, discharge location and 30-day mortality.^1,4–7^

However, despite the widespread clinical use of the cumulated ambulation score, its properties are context-specific and some psychometrical aspects still need to be assessed. Most of its psychometric properties were studied in the acute care setting, where a 3-day score is obtained from the sum of the total score of three consecutive 1-day cumulated ambulation score. However, a growing number of studies have been published using only the 1-day score, in both the acute and sub-acute phase after hip fracture or other pathological conditions.^8–10^ Nevertheless, a lack of information about the construct validity, the responsiveness and the minimal important difference of the 1-day score in older adults with hip fracture hospitalised in sub-acute phase, such as the case of admission to rehabilitation units or facilities, has been found.

The minimal important difference has been defined as a threshold for minimal within-person changes over time above which patients perceive themselves as importantly changed. Notably, the minimal important difference of the cumulated ambulation score was previously assessed in people with hip fracture hospitalised in an acute geriatric ward for a short length of stay^11^ or in a sub-acute phase^12^ and in older people hospitalised for general medical illness not related to femoral fractures.^9^ However, in these studies assessing people with hip fracture the minimal important difference was exclusively determined using distribution methods, currently considered inappropriate.^13,14^

Therefore, the aim of this study was to assess some measurement properties of the cumulated ambulation score in older adults with hip fracture admitted for rehabilitation in the sub-acute phase. Specifically, we examined the construct validity and the responsiveness through hypothesis testing and the minimal important difference using the anchor-based methods.

## Methods

Participants of this study were recruited among those consecutively admitted at the Istituti Clinici Scientifici Maugeri, Scientific Institute of Veruno (Italy), Division of Physical Medicine and Rehabilitation, between May 2020 and January 2022 as part of an interventive clinical trial (Ethics Committee approval number: #2264CE; clinicaltrials number registration: NCT04025866). Individuals were included if they were aged ≥65 years with a diagnosis of hip fracture, defined as a fracture occurring in the femoral neck (‘intracapsular’) and/or in the inter- and sub-trochanteric femoral (‘extracapsular’) area,^2^ from a low energy fall or trauma, surgically treated. Exclusion criteria were a pathological fracture, or a fracture caused by a peri-prosthetic or high energy trauma, documented contraindication to mobilisation, isolation for infection, length of stay >60 days due to clinical complications, transfer to another hospital for clinical problem or death. Written informed consent was obtained from each patient and the study complies with the Declaration of Helsinki.

### Inpatient rehabilitation programme

The main goal of inpatient rehabilitation programme was to improve the physical function, in particular the ability in performing the activities of daily living and the walking abilities. Therefore, physiotherapists provided individualised rehabilitation programme consisted of exercises for joint range of motion, muscle strength, balance, coordination, activities of daily living, gait and climbing stairs, together with education on safety and on how to use assistive devices. The progression of the rehabilitation programme was tailored based on patient's identified abilities.^15^

### Outcome measures

All the outcome measures were recorded at admission and discharge from the rehabilitation unit. Specifically, the Italian versions of the cumulated ambulation score of the functional independence measure and of the functional ambulation categories were administered by physiotherapists at the first and the last day of hospitalisation. In addition, pre-fracture functional independence related to indoor and outdoor mobility was assessed with the new mobility score.^16^

The Italian version of the cumulated ambulation score tests the basic mobility status.^17^ In particular, physiotherapists assessed through observation the ability of patients in performing the following three activities: (a) getting in and out of bed, (b) sit-to-stand from a chair with armrests and (c) indoors walking with or without an assistive device. Each of the three cumulated ambulation score activities was rated on a three-point ordinal scale, where zero is ‘not able to, despite human assistance and verbal cueing’, one is ‘able to, with human assistance and/or verbal cueing from one or more persons’ and two is ‘able to safely do, without human assistance or verbal cueing’. This results in a cumulated ambulation score total score ranging from zero (no functional mobility) to six (independent in all three activities).^4,18^

The Italian version of the functional independence measure was used to assess the individuals’ level of independence and need for assistance in performing the basic activities of daily living during the rehabilitation period.^19^ It is an 18-item ordinal scale, in which each item is scored from one (total dependence) to seven (total independence), with a total score range from 18 to 126. The scale can be subdivided into a 13-item motor subscale and a 5-item cognitive subscale. An improvement of 22 points between admission and discharge was identified as the minimal important difference for people after hip fracture.^20^

The Italian version of the functional ambulation categories is a six-point ordinal scale ranging from zero (unable to walk) to five (independent walking capacity on uneven surface and stairs) that evaluates the ambulation status by determining the amount of physical support required when walking.^21^

### Statistical analysis

The demographic characteristics of the participants and data of the outcome measures were summarised using descriptive statistics. Based on their distribution, assessed through the Q-Q plots and the Shapiro–Wilk test, data were presented as mean (standard deviation), median (25–75 quartiles) or percentage.

Based on the COnsensus-based Standards for selection of health Measurement INstruments (COSMIN) guidelines,^22^ construct validity is defined as ‘the degree to which scores of a measurement instrument are consistent with hypotheses, for instance with regard to internal relationships, relationships with scores of other instruments or differences between relevant groups’. Therefore, four a priori hypotheses were formulated for assessing the construct validity of the Cumulated Ambulation Score, which measures the construct ‘mobility’ (Table 1). Spearman correlation coefficients (rs) were used for testing the strength of correlations between the total score of the cumulated ambulation score with that of the reference scales (i.e., functional independence measure and functional ambulation categories scores at both admission and discharge). Due to the similarity in the construct to be measured, it was supposed a correlation >0.50 between the cumulated ambulation score and the other comparator instruments.

**Table 1.** Specific a priori hypotheses formulated for assessing the construct validity and responsiveness of the cumulated ambulation score.

Construct validity		Hypotheses met
*Comparison with other outcome measurement instruments*		
1	A correlation ≥0.50 will be observed between the cumulated ambulation score and the functional independence measure scale at baseline, since they measure a *similar* construct.	+
2	A correlation ≥0.50 will be observed between the cumulated ambulation score and the functional independence measure scale at discharge, since they measure a *similar* construct.	+
3	A correlation ≥0.50 will be observed between the cumulated ambulation score and the functional ambulation categories score at baseline and discharge, since they measure a *similar* construct.	+
4	A correlation ≥0.50 will be observed between the cumulated ambulation score and the functional ambulation categories score at discharge, since they measure a *similar* construct.	+
Total hypotheses met, number (%)		4/4 (100%)
Responsiveness		
*Comparison with other outcome measurement instruments*		
1	Changes in cumulated ambulation score will have moderate to large correlations (≥0.30 and ≤0.70) with changes in the functional independence measure scale.	+
2	Changes in cumulated ambulation score will have moderate to large correlations (≥0.30 and ≤0.70) with changes in the functional ambulation categories score.	+
*Comparison between subgroups*		
3	Patients classified as improved (defined as the ability to reach the minimal important difference of the functional independence measure total score) showed higher effect size respect to those classified as non-improved.	+
4	Patients classified as independent ambulators with the functional ambulation categories (score at discharge ≥3) showed higher effect size respect to those classified as dependent ambulators.	+
5	Ability of the cumulated ambulation score to discriminate between improved and not-improved patients following a rehabilitative intervention, defined as those who reached or not the minimal important difference of 22 points at the functional independence measure total score at discharge, respectively, with an area under the curve ≥0.70.	+
6	Ability of the cumulated ambulation score to discriminate between independent and dependent ambulators following a rehabilitative intervention, defined as those who obtained a functional ambulation categories score ≥3 or <3 at discharge, respectively, with an area under the curve ≥0.70.	+
Total hypotheses met, number (%)		6/6 (100%)

Responsiveness is the ability of an outcome measure instrument to detect change in the construct to be measured over time. Similar to construct validity, six a priori hypotheses were formulated for assessing the responsiveness of the cumulated ambulation score (Table 1). First, it was assumed that the change score of the cumulated ambulation score had a correlations rs ≥ 0.30 and ≤0.70 with the change score of both the functional independence measure and functional ambulation categories (hypotheses 1 and 2, Table 1). Second, based on the minimal important difference of the functional independence measure total score, participants were divided in those who experienced a true functional improvement (functional independence measure change score ≥22 points) at the end of the rehabilitative programme and those who did not (<22 points); similarly, patients were classified as independent ambulators or dependent ambulators if they obtained a functional ambulation categories score ≥3 or <3 at discharge, respectively. It was hypothesised that patients improved or independent ambulators at discharge showed higher effect of the rehabilitative treatment respect to those not-improved or dependent, respectively, as evaluated with Cohen's d effect size (hypotheses 3 and 4, Table 1). Values of effect size <0.2 were considered as trivial, 0.2–0.5 as small, 0.5–0.8 as moderate and ≥0.8 as large.^23,24^ Finally, responsiveness was examined assessing the ability of the cumulated ambulation score in identifying the subgroups of patients through the evaluation of the area under the receiving operating characteristic curve (hypotheses 5 and 6, Table 1).^25^ The improvement in the functional independence measure total score and the functional ambulation categories score at discharge were both used as anchors for constructing the receiving operating characteristic curve, with patients dichotomised based on the criteria previously reported (improved vs. not-improved, independent vs. dependent ambulators). Values of area under the curve >0.90 indicated high accuracy, 0.70–0.90 moderate, 0.50–0.70 low and 0.50 a chance result.

High levels of construct validity or responsiveness were determined when ≥75% of the hypotheses of each measurement property were confirmed, moderate when 50–75% of the hypotheses were met and low when <50% were met.^26^

Floor and ceiling effects of the cumulated ambulation score were analysed calculating the percentages of participant obtaining the lowest and the highest score at both admission and discharge. Significant effects were set at 15%.^26^

The standard error of measurement was assessed as standard deviation × √(1 − rtest–retest), where rtest–retest was the test–retest reliability of the cumulated ambulation score scale.^7^ This allowed to calculate the minimal detectable change with the following formula: 1.96 × √2 × standard error of measurement, where 1.96 is the z value (2-tailed) of the 95% confidence interval. The minimal detectable change is the smallest change in score that ensures the change is not the result of measurement error, so a valid minimal important difference should then be at least as large as the observed.^27^

The minimal important difference was determined using the anchor-based method.^28^ According to Froud and Abel,^29^ the minimal important difference was assessed using the receiving operating characteristic curve of the cumulated ambulation score. As for responsiveness, the receiving operating characteristic curves were constructed using as anchors the minimal important difference of the functional independence measure total score and the functional ambulation categories score at discharge.

All statistical analyses were carried out with Stata 13 (StataCorp LLC, College Station, Texas, United States of America).

## Results

**Figure 1.**
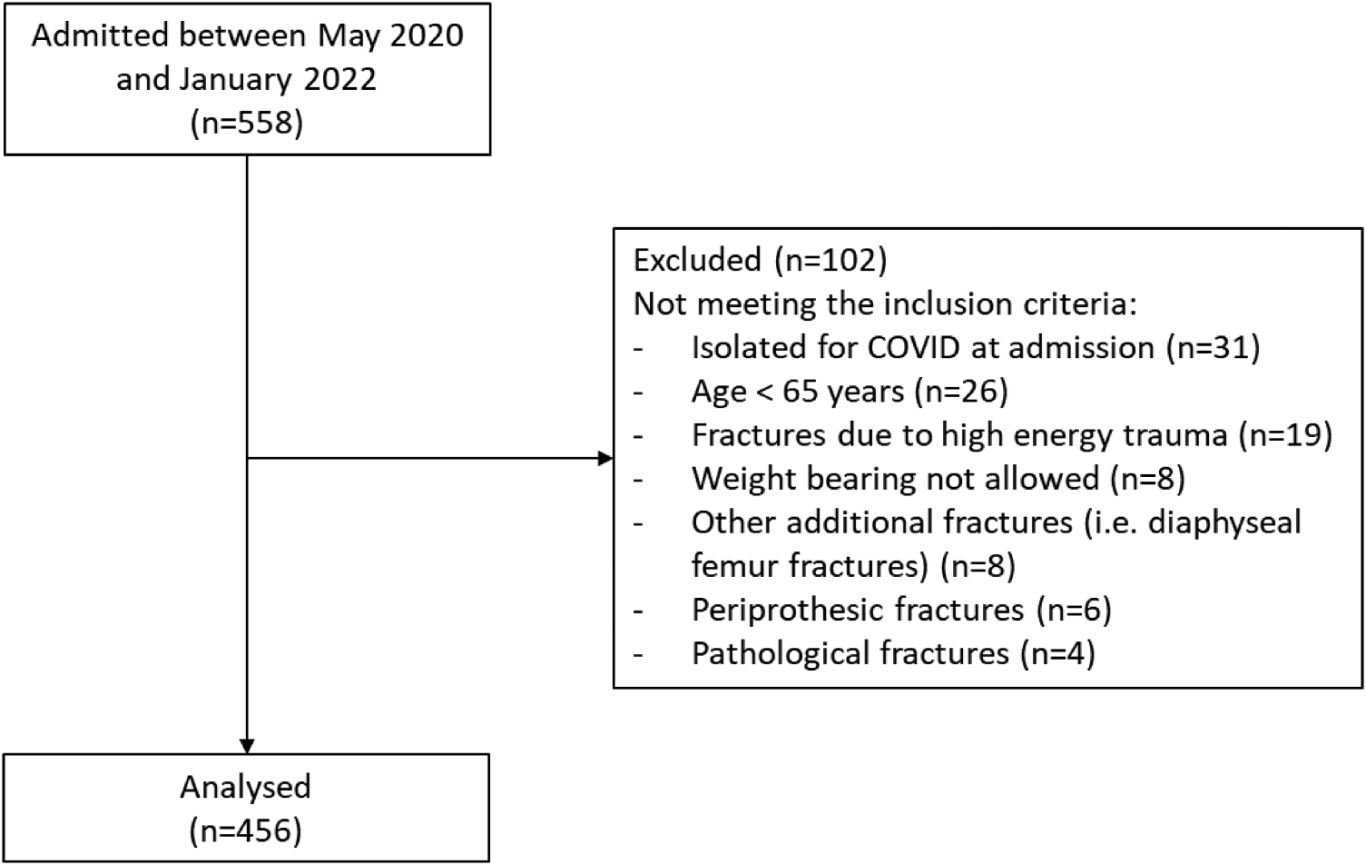
Flow chart of the study.

A total of 558 patients admitted at the rehabilitation institute were assessed for inclusion. Of these, 456 were considered eligible and recruited (Figure 1). Detailed demographic and clinical characteristics of the included patients are provided in Table 2. The mean age was 82.6 ± 7.5 years; 61.4% of patients had an inter-trochanteric fracture, while the remaining 38.6% had a neck fracture. The mean length of stay in the rehabilitation facility was 32.5 ± 7.8 days.

**Table 2.** Characteristics of recruited patients with hip fracture.

	Patients (number = 456)
Age (years)	82.6 ± 7.5
Sex, male/females (number, %)	94 (20.6%)/362 (79.4%)
Weight (kg)	64.2 ± 13.7
Height (cm)	162.7 ± 7.0
Type of fracture (number)	
Intracapsular fracture	176 (38.6%)
Extracapsular fracture	280 (61.4%)
Time from surgery to admission (days)	8.9 ± 4.4
Length of stay (days)	32.5 ± 7.8
New mobility score	6.0 (3.0–9.0)
Cumulated ambulation score at admission (total score)	3.0 (1.75–3.0)
Cumulated ambulation score at discharge (total score)	4.0 (3.0–6.0)
Functional independence measure at admission (score)	
Motor score	32.6 ± 11.1
Cognitive score	27.4 ± 6.4
Total score	60.1 ± 15.5
Functional independence measure at discharge (score)	
Motor score	55.8 ± 17.0
Cognitive score	29.2 ± 5.9
Total score	85.0 ± 21.8
Functional ambulation category at admission (score)	1.0 (0.0–1.0)
Functional ambulation category at discharge (score)	3.0 (2.0–4.0)

### Construct validity

Moderate to high correlations were found between cumulated ambulation score and functional independence measure total scores at both admission and discharge (rs = 0.68, 95% confidence interval = 0.62–0.73, p < 0.0001 and rs = 0.89, 95% confidence interval = 0.87–0.91, p < 0.0001, respectively). Similarly, the admission and discharge total score of the cumulated ambulation score highly correlated with the functional ambulation categories (rs = 0.83, 95% confidence interval = 0.81–0.87, p < 0.0001 and rs = 0.88, 95% confidence interval = 0.86–0.90, p < 0.0001, respectively).

Based on these results, four (100%) hypotheses were confirmed, indicating high levels of construct validity.

### Responsiveness

The correlations between the change score of the cumulated ambulation score and the change score of the functional independence measure and the functional ambulation categories were moderate (rs = 0.51, 95% confidence interval = 0.44–0.57, p < 0.0001 and rs = 0.54, 95% confidence interval = 0.47–0.61, p < 0.0001, respectively).

For the anchor-based method, the responsiveness of the cumulated ambulation score was assessed with the receiving operating characteristic curve. Patients identified as improved (functional independence measure change score ≥22 points) showed an improvement in the cumulated ambulation score total score of 2.5 ± 1.2 points, with an effect size of 2.48; this improvement was significantly different (p < 0.0001) from that obtained by patients classified as not-improved (functional independence measure change score <22 points), which showed a cumulated ambulation score change score of 1.4 ± 1.0 points and an effect size of 1.06. The cumulated ambulation score discriminated between improved and not-improved participants with an area under the curve of 0.75 (95% confidence interval = 0.71–0.79). According to the functional ambulation categories score, patients identified as independent ambulators at discharge had a mean increase in the cumulated ambulation score total score which was significantly different (p < 0.0001) from those identified as dependent ambulators (2.3 ± 1.2 points, effect size = 2.15 vs. 1.5 ± 1.0 points, effect size = 1.85, respectively). The area under the curve was 0.70 (95% confidence interval = 0.65–0.75). Since all the a priori hypotheses were met, the responsiveness of the cumulated ambulation score was considered to be high.

### Interpretability

**Figure 2.**
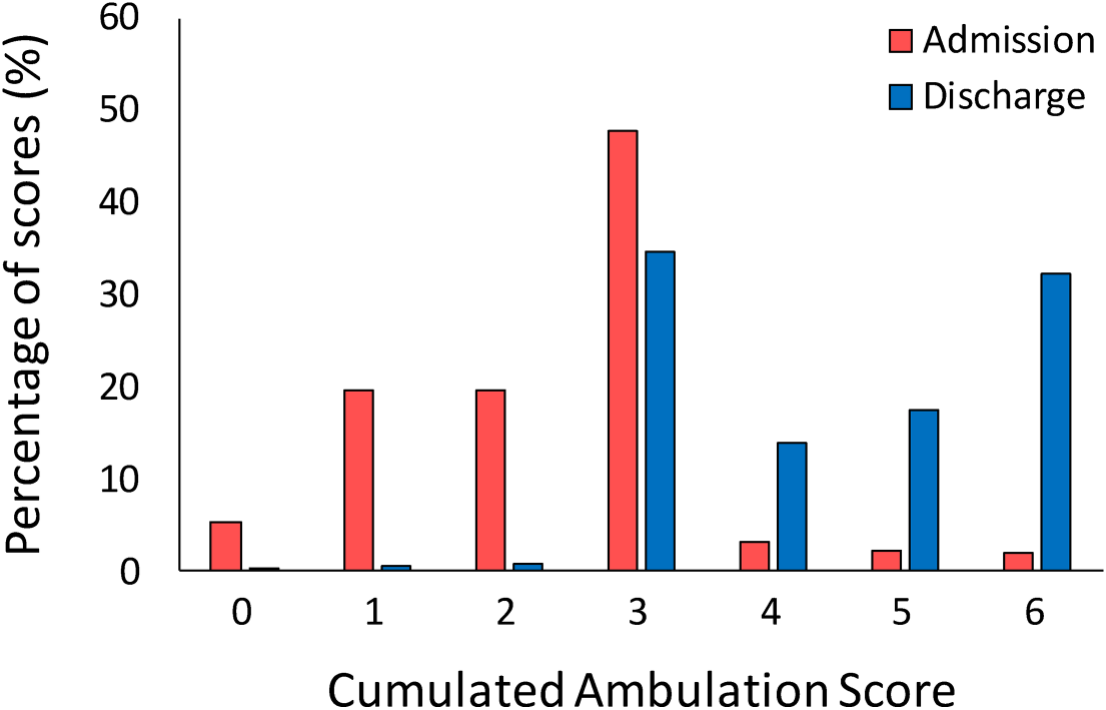
A histogram showing the percentage of patients who scored each level of cumulated ambulation score total score at admission and at discharge.

Ceiling and floor effects of the cumulated ambulation score were absent at admission, while a ceiling effect was observed at discharge, when 32.2% of the patients obtained the maximum score of six points (Figure 2).

Moreover, while at admission most of the patients (87.3%) obtained a score in the range between one and three, at discharge the highest percentage of participants (98.3%) reached a total score between three and six points, suggesting a higher independence.

The standard error of measurement for the cumulated ambulation score, calculated with the rtest–retest of previous published data,^7^ was 0.24, while the minimal detectable change was 0.67 points (equal to 11.2% of the total score).

The minimal important difference estimations performed with the two different anchors gave the same results (minimal important difference [95% confidence interval] = 2 [1.0–3.0] and minimal important difference [95% confidence interval] = 2 (1.3–2.7) points, respectively, for the functional independence measure and the functional ambulation categories scores). Since the estimated results were higher than the minimal detectable change of 0.67, a mean change of at least two points was identified to be the minimal important difference of the cumulated ambulation score.

Based on the minimal important difference of two points, 309 participants (67.8%) showed a clinically important difference between admission and discharge. Of those who did not reach an improvement of at least two points (number = 147, 32.2%), 19 patients were not allowed to reach the minimal important difference of two points having a cumulated ambulation score total score at admission between five and six.

## Discussion

This study provided a comprehensive examination of some psychometric properties of the cumulated ambulation score in older adults with hip fracture hospitalised in the sub-acute phase for rehabilitation. The construct validity and the responsiveness were both high according to the hypothesis testing. An improvement of two points at the end of rehabilitation programme was clinically important. A ceiling effect was found only at discharge, while no floor effect emerged at both admission and discharge.

To our knowledge, this was the first study which assessed both the construct validity and the responsiveness of the cumulated ambulation score using the hypothesis testing procedure, as suggested by the COSMIN guidelines.^22^ The construct validity was satisfactory, with 100% of the a priori hypotheses met. High correlations between cumulated ambulation score and both the functional independence measure and the functional ambulation categories at admission and discharge were found. These results were expected since the cumulated ambulation score, which assesses the basic mobility independence, was correlated with instruments assessing patients’ independence in performing activities of daily living and in walking. Even without considering the hypothesis testing, the construct validity was previously evaluated in people with hip fracture and in those with other heterogeneous diseases through the analysis of correlations existing between the cumulated ambulation score and other measures assessing independence and ability to perform activities of daily living.^11,12,17,20,30^ Responsiveness was assessed with both the distribution- and anchor-based methods. The good correlations found between the change score of the cumulated ambulation score and the change score of the other instruments reflected the ability of the cumulated ambulation score in detecting changes over time. As expected, the cumulated ambulation score increased significantly with increasing functional independence measure and functional ambulation categories scores, meaning an improvement in patients’ health status. These results are in line with previous studies reporting high effect size of the cumulated ambulation score after a rehabilitative programme, at both acute and sub-acute phase post-surgery.^11,12^ Moreover, receiving operating characteristic analysis highlighted the good capability of cumulated ambulation score change in accurately classifying subjects improved and independent ambulators from those not-improved or dependent ambulators. However, being the lower bound of the 95% confidence interval of the area under the curve having the functional ambulation categories score as anchor below the cut-off of 0.70, it may be argued an uncertainty in the estimate of responsiveness. This may be related to the fact that gait is measured by only one item of the cumulated ambulation score.

Regarding interpretability, a ceiling effect was found at discharge, with about 32% of patients who reached the maximum score on the cumulated ambulation score. These results agree with those of two previous studies reporting a ceiling effect of the cumulated ambulation score.^9,12^ In addition to identifying the possible limitation of the cumulated ambulation score, these findings highlight the importance of selecting clinical scales able to detect improvement based on the abilities of the patients being assessed. Therefore, although recent guidelines strongly suggest the use of the cumulated ambulation score in both the acute and sub-acute settings following hip fracture,^1,2^ this measure seems to be accurate when used the first days after surgery and/or in low-performing patients; conversely the cumulated ambulation score should be replaced with other more demanding measures when assessing patients in the sub-acute setting with good mobility status after rehabilitation. Finally, to our knowledge, the minimal important difference of the cumulated ambulation score has been already evaluated in people with hip fracture in the acute and sub-acute phase, and in older individuals hospitalised for general medical illnesses.^9,11,12^ All these studies reported a minimal important difference below the score of one point. This is probably because the statistical methods used were not appropriate (distribution-based instead of anchor-based methods)^13,14^ or because the mean cumulated ambulation score at admission was near the maximum score of six points. While questioning the usefulness of recommending a change between 0.5 and 0.8 as clinically important even if the scale has integer intervals, we believe that clinicians and researchers should consider the minimal important difference value more adequate based on the population considered. The minimal important difference of two points estimated in this study was able to identify about 68% of patients which obtained a clinical meaningful change at discharge from the rehabilitative institute. In the interpretability of this result, it is of crucial importance to note that patients enrolled in this study were older adults with a hip fracture due to low energy falls and with low level of independence at admission.

Main strengths of this study include the large sample size and the robust methodology used for assessing the measurement properties of the scale. Validity and responsiveness were established with hypothesis testing, in accordance with the internationally recognised COSMIN guidelines^22,28^; moreover, the minimal important difference was estimated using the recommended anchor-based method.^28,31^ Conversely, the main limitation of this study is the fact that the anchors used for estimating the minimal important difference were all clinical measures, and not patient-reported global rating of change. Nevertheless, it has been argued that, in the clinical research context, the global ratings of change are strongly biased by health status at the time of assessment, do not adequately or consistently correlate with functional change, and are therefore not considered valid measures of change over time.^32^

In conclusion, the cumulated ambulation score showed good levels of construct validity and responsiveness, with this measure being able to identify change after rehabilitation in the sub-acute phase. A minimal important difference of two point has been found to be accurate for assessing clinical meaningful change in older adults with sub-acute hip fracture at the end of a rehabilitation programme.

**Clinical messages**

- An improvement in the cumulated ambulation score of at least two points at the end of rehabilitation programme indicates a clinical meaningful change in people with hip fracture.
- Validity and responsiveness of the cumulated ambulation score were high, supporting its effectiveness in tracking functional improvements over time.
- Clinicians should be aware of ceiling effects when using the cumulated ambulation score in sub-acute settings.
